# Do Nobel Laureates Create Prize-Winning Networks? An Analysis of Collaborative Research in Physiology or Medicine

**DOI:** 10.1371/journal.pone.0134164

**Published:** 2015-07-31

**Authors:** Caroline S. Wagner, Edwin Horlings, Travis A. Whetsell, Pauline Mattsson, Katarina Nordqvist

**Affiliations:** 1 Battelle Center for Science and Technology Policy, John Glenn College of Public Affairs, The Ohio State University, Columbus, Ohio 43210, United States of America; 2 Rathenau Institute, The Hague, Netherlands; 3 Department of Learning, Informatics, Management and Ethics (LIME), Tomtebodavägen 18 A, Karolinska Institutet, Stockholm, Sweden 171 77; 4 Nobel Museum, Stortorget 2, Gamla Stan, Box 2245, 103 16 Stockholm, Sweden; State University of New York, Oswego, UNITED STATES

## Abstract

Nobel Laureates in Physiology or Medicine who received the Prize between 1969 and 2011 are compared to a matched group of scientists to examine productivity, impact, coauthorship and international collaboration patterns embedded within research networks. After matching for research domain, h-index, and year of first of publication, we compare bibliometric statistics and network measures. We find that the Laureates produce fewer papers but with higher average citations. The Laureates also produce more sole-authored papers both before and after winning the Prize. The Laureates have a lower number of coauthors across their entire careers than the matched group, but are equally collaborative on average. Further, we find no differences in international collaboration patterns. The Laureates coauthor network reveals significant differences from the non-Laureate network. Laureates are more likely to build bridges across a network when measuring by average degree, density, modularity, and communities. Both the Laureate and non-Laureate networks have “small world” properties, but the Laureates appear to exploit “structural holes” by reaching across the network in a brokerage style that may add social capital to the network. The dynamic may be making the network itself highly attractive and selective. These findings suggest new insights into the role "star scientists" in social networks and the production of scientific discoveries.

## Introduction

The unique status, posture, and prestige of the Nobel Prize have made it an enduring subject in the sociology of science [1–9]. According to Alfred Nobel’s will, the Prize should be awarded to those who “shall have conferred the greatest benefit to mankind.” In Physiology or Medicine the Prize can be shared by up to three scientists each year who have made the most important discovery in their domain. This study explores and identifies the collaborative behavior of scientists embedded within research networks in Physiology or Medicine. Our focus is not on predicting Prize winners or explaining the impact of the Prize on the winner. Rather, we explore whether Prize winners have distinctive collaboration behavior and whether they are embedded more within distinctive research networks than non-Prize winning contemporaries.

In seeking to understand the driving forces behind exceptional scientific accomplishment, the literature on Nobel Laureates has explored their productivity, impact, and collaboration [8], as well as the effects of age on productivity and creativity [5, 10]. Still other studies have broadly analyzed the impact of other prizes such as Howard Hughes award [11], and Fields Medals [12]. However, Harriet Zuckerman [8] first raised questions about the differences in productivity patterns of Nobel Prize awarded scientists, noting specific strategies and behaviors that differentiate Laureates from non-Laureates. Our sample of Nobel Laureates begins where Zuckerman’s ended at the end of the 1960s. This research project raises similar questions but also develops data using different design elements and methods of analysis on a sample of Prize winners in Physiology or Medicine and a matched comparison group of highly productive scientists.

A review of the literature reveals no consensus on the distinguishing features between exceptionally creative scientists who have and have not won the Nobel Prize. Certainly many differences are evident between Nobel Laureates and average scientists. However, comparing Laureates to average scientists is not revealing: we compare them to other similar scientists in order to produce more meaningful insights into their characteristics and behaviors. Several studies have employed matching group designs to address such questions. On the question of productivity, Zuckerman [8] finds a group of American Nobel Laureates in the sciences to be more productive than a matched group. Garfield & Welljams-Dorof [6] examine science prize winners with a comparison group and find productivity differences to be slight, but with lower overall productivity among the Nobel Laureates in the sciences. Hirsch [13] finds that some Nobel Prize winners (Physics only) had lower productivity than other successful non-Laureates, although he noted a broad range of productivity patterns among highly cited scientists. Studies on citation impact consistently show the Nobel Laureates to be more highly cited than the average scientist; however, when compared to other productive scientists, the record is mixed, especially before winning the Prize. Sher & Garfield [14] find that, even before winning the Prize, the 1962 Nobel Laureates in Physics, Chemistry, and Physiology or Medicine had garnered significantly higher citation counts than the average scientist in the Science Citation Index (SCI). Garfield & Welljams-Dorof [6], also using SCI, calculated the publication record of eight Laureates. The results show higher citation strength on average (28.9% higher) than an elite group of non-Laureates, also before winning the Prize. Hirsch [13] also finds a broad range of citation strength, and no particular pattern among Nobel Prize winners.

The record is also mixed on the collaborative patterns shown by the Nobel Laureates. Zuckerman [8] finds that the American Nobel Laureates in science showed a greater propensity to collaborate with other Laureates than did a matched group. She also notes that, as young scientists, Laureates were more likely to be the first author or sole author on publications, but over time, this distinction fades. Price & Beaver [15] show that elite scientists are more likely to collaborate. In a study of highly-productive, commercially-oriented bioscientists, Zucker and Darby [16] report that “star bioscientists were very protective of their techniques, ideas, and discoveries…” (p. 12709) and thus are *less likely* to collaborate; the “stars” were more likely to appear as the last author rather than the first author on publications, which is most common for senior scientists within the biomedical fields. This leaves unanswered a number of questions about productivity, impact, and collaboration behavior of the Nobel Laureates.

To answer these questions, we compiled data on the publishing and co-authoring patterns of Nobel Laureates and a matched group of elite scientists in biomedicine. We add to the existing literature by creating a matched comparison group of non-Laureates who appear to be “of Nobel class” [6] matched by the domain of biomedicine, h-index, and year of first publication. Further, we apply network analysis to examine the relational dynamics of collaboration among the two groups embedded within their wider research collaboration networks. We offer insights into the dynamics of collaboration among top scientists in an effort to provide guidance to those policymakers and research managers who seek to encourage and support exceptional creativity in scientific research.

## Data Collection, Design, & Methods

First, meta-data for all articles, reviews, notes, and letters of 68 Nobel Laureates in Physiology or Medicine winning the Nobel Prize between 1969 and 2011 were identified and extracted from the Web of Science bibliometric database using the author search feature (N~15,000). Many Nobel Prize winners have common names, and disambiguation quickly became an obstacle to a comprehensive data set. Therefore, scientists were included only if a first and middle initial were obtainable (e.g., Murray, J.E. rather than Murray, J.), or, if a name was unusual enough to ensure a limited number of false positives. In some instances the database allowed for accurate identification of records through its "distinct author record set" feature. To avoid false positives from common names, the sample of Laureates was limited to 68 out of 101 who were awarded the Nobel Prize between 1969 and 2011. A sight check was also conducted by comparing publications to curriculum vitae.

A similar procedure was conducted for 68 matched non-Laureate scientists: Each of the 68 Nobel Laureates was matched with one of 68 comparison group members using three criteria: 1) common field of research (“biomedicine” as tagged by Web of Science); 2) year of first publication (range 1922–1992 for Laureates; 1921–1994 for non-Laureates); and 3) h-index (range 7–153 for the Laureates; 17–160 for non-Laureates; average 72 in both).

The research area “biomedicine” is quite broad, and so title and reference co-occurrence analyses were conducted to test whether the two groups published on the same topics (for the method, see Horlings & Gurney [17]). First, we combined all the publications of Laureates and non-Laureates into one database. Then, we calculated the similarity between each pair of publications in terms of shared words. Similarity is measured as a Jaccard coefficient. The result is a network of publications (nodes) and their similarities (edges). The Louvain clustering algorithm of Blondel et al. [18] was used to identify distinct clusters. Of all the topics (clusters of publications) 69% contain either a Laureate or a non-Laureate, while 31% contain both a Laureate and non-Laureate. To provide some context, Boyack et al. [19] identified between 20,000 and 30,000 topical clusters among 2 million publications in the biomedical data base Pub-Med. The topical clusters identified in our sample comprise approximately one percent of this total. Thus, the chance of any random set of non-laureates matching 31% of topics in which Laureates are active is very low.

The h-index, developed by Jorge Hirsch [13], was chosen because it balances productivity and citation impact. Hirsch noted that an h-index of 40 characterizes outstanding scientists, and that an h-index of 60 shows “truly unique” characteristics. The h-index is calculated in Web of Science’s records analysis feature. H-index appears to be robust against disambiguation problems among elite scientists, since false positive records are very unlikely to be as highly cited as those of an elite scientist.

Year of first publication was chosen as an indicator of the beginning of the scientist’s research career. Matching on year of first publication is critical because it places the careers of the two groups in the same era. This is important because there is a significant increase in the general scientific coauthorship rate throughout the time frame of our study. Using year of first publication also may provide a better match than age, since scientists begin their careers at different ages.

Next, bibliometric statistics were calculated at two levels: the author level (Table 1), and the publication level (Table 2). The Wilcoxon rank sum procedure was used to test for statistically significant differences between the two groups. For the Laureates, generalized linear model (GLM) with Poisson distribution and logistic regression were used to examine authorship patterns before and after winning the Nobel Prize (Table 3). Counts are made regardless of author order (first, middle, or last author). This is in contrast to other studies [6] which used only first authors. Wren et al. [20] found that first and last authors have the greatest responsibility for leadership of research projects in the biomedical sciences, but we did not limit collection or analysis to those positions. Sole authors were also included as a major focus of this research.

**Table 1:** Descriptive Statistics at Author Level for Laureates and Non-Laureates.

Groups	Measures	Records	Total Distinct Coauthors	Percent Sole Author	Percent First Author	Percent Last Author
**Laureates**	Mean	222	344	16	14	47
**N = 68**	Median	180*	253*	10*	10	50
	St.Dev.	194	326	17	10	18
**Non-Laureates**	Mean	306	455	8	15	43
**N = 68**	Median	255*	321*	5*	11	45
	St.Dev.	198	402	8	13	16

* = P<0.05—Wilcoxon Rank Sum Test of Median Difference between independent samples. The total number of sole authored papers was divided by the total number of papers for each primary author. The same was done for first author percentages, except sole authored publications were first subtracted. Authors were disambiguated with VantagePoint Software and then aggregated for each primary author.

**Table 2:** Descriptive Statistics at Paper Level by Laureates and Non-Laureates.

Groups	Measures	Times Cited	Number of Authors per Co-authored Paper	Number of Nations per Paper	Percent of International Papers
**Laureates**	Mean	117	5.0	1.3	23
**N = 14,595**	Median	45	4.0	1.0	0.0
	St.Dev.	329	7.0	0.7	42
**Non-Laureates**	Mean	90	5.1	1.3	21
**N = 20,580**	Median	32	4.0	1.0	0.0
	St.Dev.	317	4.6	0.8	41

All differences are statistically significant at the P<0.05 level due to large sample size. Number of Authors is calculated after eliminating sole authored records reducing the sample size—Laureate N = 13,104; Non-L. N = 19,221. Nation data is missing for a large number of older papers—Laureates N = 9,165; Non-L. N = 13,076. The total number of international papers (2+) was divided by the total number of papers with country data available to calculate international percent.

**Table 3:** The Effect of the Prize on Laureates’ Authorship Patterns.

Time Period	Measures	Number of Authors per Co-authored Paper	Percent Sole Author
**Pre-Prize**	Mean	4.4*	11.0*
**N = 9620**	Median	4.0	0
	St.Dev.	5.0	31.0
**2 Years Post-Prize**	Mean	6.2*	17.0*
**N = 259**	Median	5.0	0
	St.Dev.	7.0	0.37
**Total Post-Prize**	Mean	6.4*	9.0*
**N = 5091**	Median	5.0	0
	St.Dev.	10.0	29.0

* = P<0.05 by GLM with Poisson distribution & logit

Next, the names of all authors in the co-authored publications data sets were disambiguated to reduce error. Author disambiguation is one of the main challenges in constructing coauthor networks. There are two problems with the names of authors in indexes of academic publications such as the Web of Science. First, author names may occur in different spelling variants, related to initials, hyphens, spelling errors, and name changes resulting from marriage. These features can produce node duplication that fragments the coauthor network; it can be particularly acute with highly connected authors such as Nobel Prize winners. Second, different authors may have the exact same name and/or last name and first initial. This results in conflation of what should be separate authors. Without author disambiguation many coauthor network measures are unreliable. We disambiguated the records using various methods. For the descriptive bibliometric statistics in Table 1, records were disambiguated using the data mining software Vantage Point. For the remaining analyses a second set of methods were used. We applied the principles of Gurney et al. [21] by first calculating the similarity between authors in the co-occurrence of title words and coauthors, the only metadata that occur in all articles. The second indicator concerns proximity in the dataset. Raw Web of Science data were parsed into a relational database using the SAINT toolkit [22]. Similar author names that were processed in each other’s vicinity are more likely to refer to the same person. These two indicators provided a heuristic for manual disambiguation based on similarities in spelling [21].

After disambiguation, the resulting records allowed us to create three different types of networks. The first analysis compared the entire Laureate coauthor network with the entire non-Laureate network. The second analysis compared only the primary author networks from both groups, i.e. the Laureates and the non-Laureates without coauthors. The third combined the two groups. The free network analysis software, Gephi, was used to analyze and visualize the networks, providing the basis of comparison between the Laureate and non-Laureate networks.

### Authorship differences between the Laureates and non-Laureates

We compared the publication patterns between the Laureates and non-Laureates using data aggregated at the author level, shown in Table 1. In terms of numbers of publications, the Nobel Laureates produced on average significantly fewer papers over the course of their careers than the non-Laureates supporting Garfield and Welljams-Dorof [6] and Hirsch [13]. Laureates also had fewer coauthors across their career. Further, the Nobel Laureates produced a significantly higher percentage of sole-authored papers than the non-Laureates both before and after winning the Nobel Prize (see also Table 3). The two groups exhibited roughly identical percentages of papers where they appear as the first author or last author.

Next, the data were aggregated at the paper level to conduct descriptive statistical analyses for the two groups with results shown in Table 2. The data show that the Laureates accumulated many more citations per paper (times cited) than the non-Laureates, supporting Garfield & Welljams-Dorof [6], even with matched h-indexes. The data show no meaningful differences between the groups in the average number of authors per co-authored paper, the average number of nations per paper, or the percentage of international connections.

Then, we examined whether winning the Prize had an effect on coauthorship and sole authorship rates, which could explain the difference observed between Laureates and non-Laureates. Table 3 shows that the Laureates increased the average number of authors per co-authored paper after winning the Prize, but on average they are no more collaborative than the non-Laureates. Further, Laureates show a spike in sole-authored publications in the two years after winning the Prize, perhaps due to the increasing request to write review articles. But after two years the number of sole-authored publications quickly returns to pre-Prize levels. Against expectations, winning the Prize appears to have an insignificant effect on the higher rate of sole-authored publications observed among the Nobel Laureates—that tendency exists both before and after winning the Prize.

It is well documented that science as a whole has been growing exponentially and that coauthorship and international collaboration have also risen at spectacular rates [23]. The Laureates and non-Laureates follow this trend: Both the Laureates and the non-Laureates show a striking rise in productivity and collaboration rates over the past few decades. The data show a notable increase in the numbers of authors and nations represented per paper, again consistent with science as a whole, but neither group diverges from the other in productivity growth or collaboration patterns. Also reflecting changes in science, more recent papers include some with more than 100 authors. Out of around 35,000 articles in our data set, 20 have above 100 authors. These outliers are distributed roughly equally between the Laureates and non-Laureates.

In summary, the Laureates produced fewer papers than the non-Laureates, but they have a higher average impact per paper (overall the two groups have equivalent impact). In contrast to Zuckerman’s finding [8], the Laureates have a lower number of coauthors across their careers. The two groups are equally collaborative at the paper level both nationally and internationally. The Laureates produced more sole-authored papers both before and after winning the Nobel Prize. In the two-year period after they win the Prize, there is a spike in sole-authored papers, but this is only a temporary state which quickly reverts to the original pattern. After winning the prize the Laureates show on average higher numbers of coauthors per paper. In keeping with trends in science overall, the Laureates have become more productive and more collaborative over the decades.

## Network Analysis Results

**Fig 1:**
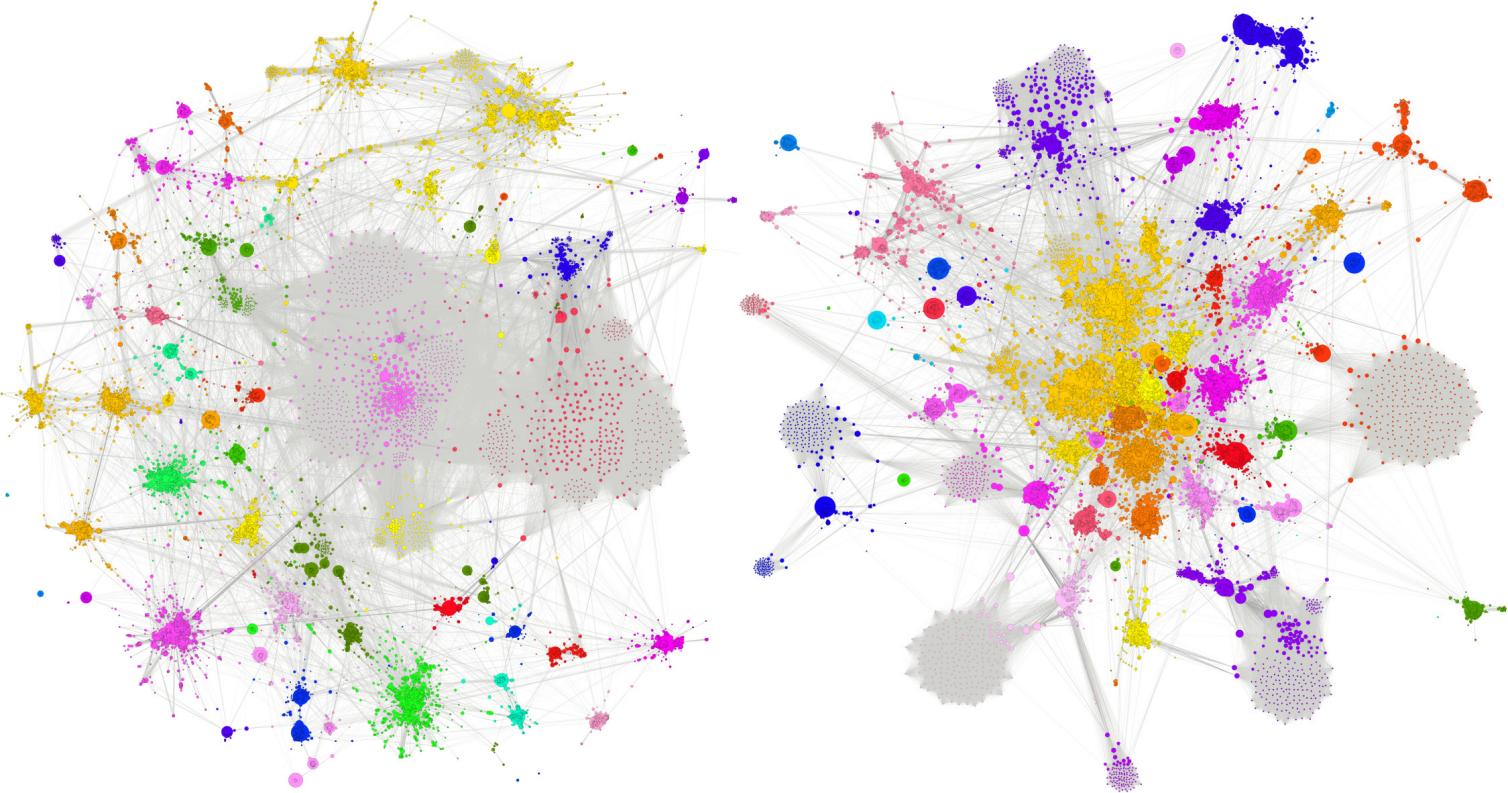
Nobel Laureate Coauthor Network (left) and Non-Laureate Coauthor Network (right). Network graphs were produced in Gephi, using the Force Atlas 2 layout, a variant of the Fruchterman-Reingold algorithm with stronger clustering. Linlog mode was used with scaling set to 0.2 and gravity to 10. After applying the Force Atlas 2 layout, Noverlap was used to prevent nodes from overlapping [27]. Node coloring is based on modularity class, identified using the Blondel et al. [18] algorithm.

**Fig 2:**
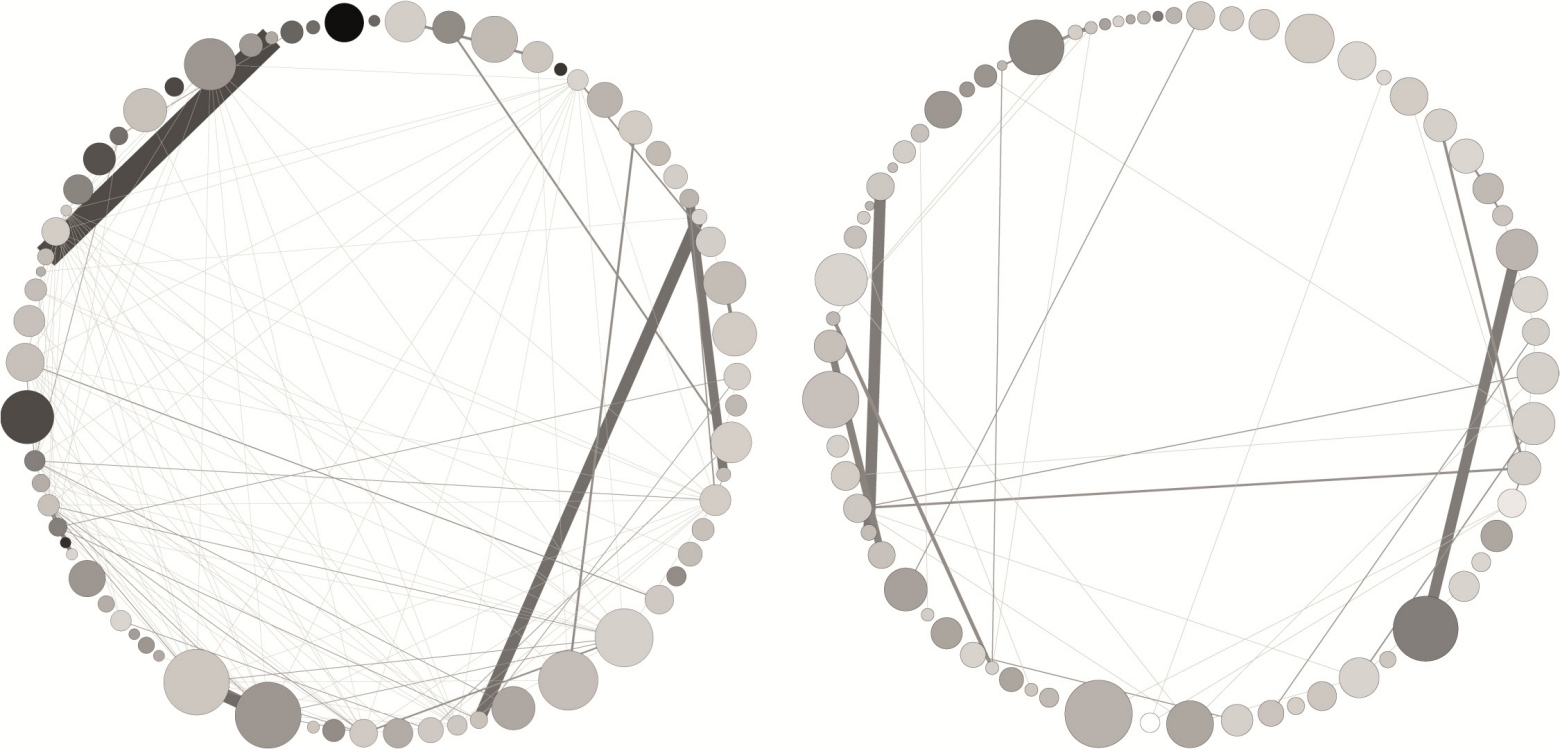
Circular Hierarchy of Coauthor Relations Among Nobel Laureates (left) and Non-Laureates (right). Authors are arranged clockwise starting from top center in ascending order from the year of first publication. The size of the nodes indicates the average number of citations received per paper produced by each scientist. Color of the nodes indicates the percentage of sole-authored publications in lifetime output, ranging from 0% (white) to 78.6% (black). Thickness of the edges indicates the number of co-authored publications.

**Fig 3:**
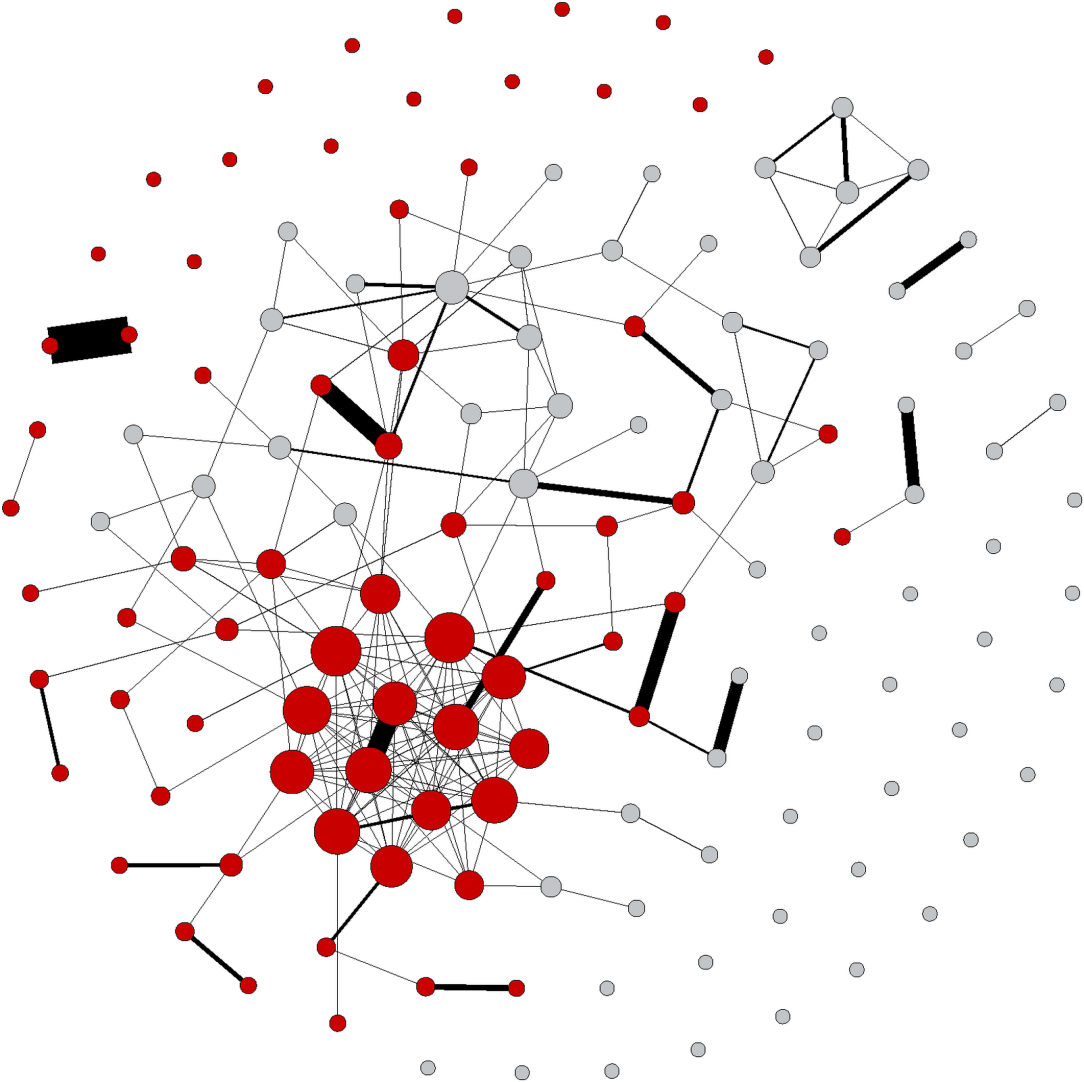
Coauthor Relations among Nobel Laureates (scarlet) and non-Laureates (grey). Fruchterman-Reingold layout was used. Node size is based on degree. Laureates are scarlet, and non-Laureates are grey. Edge size represents weight.

The publication data allowed us to extract and compare coauthor networks to explore collaborative patterns. Three types of networks were constructed for the analysis: 1) comparison of the Laureate coauthor network with non-Laureate coauthor network, *including all coauthors* (Figs 1 and 2) comparison of the Laureate coauthor network with the non-Laureate coauthor network *without coauthors*, *i*.*e*. *only primary authors* (Figs 2 and 3) a combined Laureate/non-Laureate coauthor network *without coauthors*, *i*.*e*. *only primary authors* (Fig 3). Network measures were derived for average degree, average clustering coefficient, modularity (community structure), number of communities, density, and the average path lengths.

The most impressive finding is that the Laureate network including all coauthors has significantly greater connectedness than the network around the non-Laureates. Table 4 shows that the average degree for the Laureate network is higher than for the non-Laureate network, which suggests that on average the authors are more ‘popular’ and indicates the possibility of higher social capital [24]. The degree of an author is a measure of the number of ties connecting her or him to other authors in the network. The average degree measures the structural cohesion of the coauthor network as a whole [25]. Average degree demonstrates that authors in the Laureate network (despite having fewer members) have realized many more ties than those in the non-Laureate network. The density measure further supports this observation by showing that the Laureate network is twice as dense as the non-Laureate network. Network density measures the proportion of all connections made, out of all that could be made, for the network as a whole. Modularity for the Laureate network is lower than in the non-Laureate network. Modularity measures the extent to which the network is partitioned into “communities of densely connected nodes, with the nodes belonging to different communities being only sparsely connected”[18]. Similarly, the number of communities counted in each network shows that the Laureate network has significantly fewer distinct communities, also indicating that the Laureate network is more interconnected and less clustered. The two networks with coauthors have roughly equal path lengths of 4. A path length measures the average number of steps from one place in the network to any other place in the network; in other words, both networks allow coauthors to be four “handshakes” away from each other should they wish to reach out to new connections [25]. When coauthors are removed from the analysis, the small world nature of the Laureates network is even more evident, with the average path length dropping below 3 “handshakes.”

**Table 4:** Comparative Analysis of Laureate and Non-Laureate Networks with and without Coauthors.

Groups	Number of Authors	Avg. Degree	Modularity	Density	Avg. Clustering Coefficient	Number of Communities	Avg. Path Length
**Primary Authors and All Coauthors**							
**Laureates**	20649	32.940	0.795	0.002	0.870	40	3.996
**Non-Laureates**	27789	23.115	0.914	0.001	0.863	55	4.057
**Primary Authors Only**							
**Laureates**	68	3.912	0.656	0.058	0.459	29	2.962
**Non-Laureates**	68	1.118	0.828	0.017	0.441	39	3.374

Network measures for “primary authors and all coauthors” are represented visually in Fig 1; Measures for “primary authors only” represented in Fig 2. The degree distributions are non-normal and highly right skewed. Therefore, the non-parametric independent samples Mann-Whitney test was used to test for statistical significance. Results for “primary authors and all coauthors” are U = 284308736, Z = -1.710, Sig = 0.087. Results for “primary authors only” are U = 1712.5, Z = -2.747, Sig = 0.006. Numbers for “primary authors only” were calculated after filtering for nodes and edges between primary authors separately for each group.

If compared to a random network, both the Laureate and non-Laureate networks have high clustering coefficients and short path lengths—features common to small world networks [26]. ‘Small worlds’ facilitate rapid transmission of information across a network. The small world-ness can be gleaned from Table 4 where the groups show high clustering and connectivity and a small number of intermediaries (path lengths). The connections are conduits for information flows, but the measures suggest that the Laureate network has many more realized connections (or links) across the network (average degree), creating enhanced opportunity for bridging to new ideas, methods and technologies. The non-Laureates show higher modularity with fewer links between clusters. Thus, the non-Laureate network could be interpreted as more bonded, but with less bridging than seen in the Laureate network.

Fig 1 shows the two networks with all the Laureates and their coauthors (left), and all the non-Laureates and their coauthors (right). The networks represent co-authorships in more than 15,000 publications over more than 70 years for each network. Each line represents a co-authoring event. The non-Laureate network is visibly more modular with less interconnection between communities, revealing bonding within tight community structures.

Another key finding from the network analysis is that the Laureates are highly likely to be connected to other Laureates, beyond the collaboration of Laureates winning the prize together. Twelve pairs of the Nobel Laureates won the prize in the same year for work conducted together. Accordingly, we tested for a potential selection bias in the Laureates data by removing the links between the 12 pairs from the Laureate coauthor network (represented in Fig 2). Even after removing those winning the Prize together, the Laureate network retained a much higher level of interconnectivity than the non-Laureate network. Thus, supporting Zuckerman [8], network analysis reveals that the Laureates are more connected to one another than the non-Laureates. In other words, the Laureates are more likely to have worked with other Laureates over the course of their career. Their coauthors are also more likely to have co-authored with each other. Fig 2 shows the networks as circular graphs of coauthor relations among Laureates (left) compared to the non-Laureates (right).

Fig 2 shows the greater interconnectedness of the Laureate network, i.e. connections between Laureates themselves. The extent of connectedness suggests that ideas, technology, and resources may spread much more easily among the Laureates, as in a “viral” network. The lower modularity measure and smaller number of communities (Table 4) suggests opportunities for faster diffusion of knowledge within the Laureate network. Monge and Contractor [24] report that less centralized networks are more efficiently structured for tasks that require creativity and collaborative problem solving. We can infer from this measure that ideas, new skills, and new technologies are more accessible in the Laureate network.

The extent of connectivity among the Laureates is even more notable when the two groups of authors are placed into a single network. Fig 3 shows the combined coauthor relations among the Laureates and the non-Laureates (some of whom have also co-authored). The figure shows the dominance of the Laureates (scarlet) in terms of centrality, as well as the intensity of their interconnection to one another, compared to the non-Laureates (grey).

In summary, the Laureate networks reveal significant differences in social cohesion than the non-Laureate networks. The Laureate networks appear to be more interconnected, with many more bridging opportunities realized. They are less modular (tightly bonded into communities) and could be considered more open to the possibility of new connections; their lower modularity suggests the potentiality for greater flexibility, or reconfigurability. The Laureate networks appear to be highly attractive to other ambitious collaborators, suggesting the operation of the Matthew effect noted by Azoulay et al. [11].

## Discussion

A relevant question for this study has been: does the Nobel network have higher social capital? We found that appropriately matched groups showed significant differences in relevant measures. The non-Laureates tend to be more productive and they have far more coauthors over the course of their careers. From these measures, we might conclude that appropriately matched non-Laureates make more attractive collaborators than the Laureates. However, despite absolute numbers, the two groups have very similar rates of collaboration per paper, both domestic and international (average number of coauthors and nations per paper). We also found very similar rates of first and last authorship, indicating that the groups are both highly visible in terms of name order recognition and demonstrate high levels of leadership, i.e. first or last author position. These similarities would seem to suggest that appropriately matched non-Laureates exercise very similar levels of social capital to their Prize winning counterparts. Indeed, at this level of analysis, the similarities outweigh the differences. Ending the analysis here would suggest very few differences.

But that is not the whole story. The bibliometric analysis revealed two key differences. First, Laureates are more highly cited, despite roughly equivalent one-to-one matching by h-index. This may indicate that Laureates focus on fewer, higher quality publications. In terms of career-long calculations, their h-indices may be equal across the groups, but the quality of work by the Laureates appears to be higher. Second, Laureates produce a significantly higher rate of sole authored publications. We found this to be the case both before and after winning the Prize. Greater sole authorship could potentially increase their visibility in the field and to the scientific community broadly. The sole authorship may indicate that Laureates are in the forefront of change in their research field and therefore produce more reviews—a publication form that normally is connected with sole authorship. They may also exercise greater discretion when using their social capital, i.e. they may be more selective in the research that they publish, and in their choice of students and coauthors.

The Laureates appear to operate within and influence networks in ways different from peers, which may reflect a distinction in the ways that Laureates manage social capital within their coauthor network. The greater structural cohesion of the Laureate network, which features higher average degree, greater density, lower modularity, and fewer distinct communities, is consistent with Burt’s [28] description of social capital in networks and the bridging of structural holes. Burt [28] has shown that holding a key position in a network can be an asset equivalent to social capital. Social capital is simply that better-connected people enjoy greater benefits from participating in the network. This may be the case with the Laureate network. Further, we find that the Laureates are far more likely to have co-authored publications with other Nobel Laureates. It may be that the quality of the work being done within the Laureate network attracts the highest quality collaborators, or, it may be that collaborators gain attention because they are working with Laureates and thus become more likely to win the Prize themselves.

The greater structural cohesion of the Laureate network could also be interpreted using contagion theory [29], in which opportunities for quick diffusion of information result from readily-made contact. However, elite scientists do not necessarily wish to “catch” the ideas of others so much as to differentiate themselves and develop novel and innovative solutions [30]. The Laureates may broker connections across structural holes and therefore know about and access more rewarding, information-rich activities. Burt [28] suggests that those who bridge structural holes monitor information more efficiently than others, and this feature may enable the Laureates to better differentiate their work within the research network, giving them an advantage and providing greater overall social capital to the network, which provides a virtuous cycle of attraction similar to the Matthew effect [11].

With the knowledge in hand of what other creative scientists are researching, highly skilled actors are able to efficiently exploit structural holes by bridging communities. Monge and Contractor [24], like Burt, suggest that those who successfully bridge structural holes in communications networks attain a competitive advantage over others and also enhance their own structural autonomy because they can control the information flows. In order to identify structural holes or new knowledge flow opportunities, actors must know their networks very well. The Laureate network is structured in a way that allows more interaction and visibility, and therefore more knowledge about the work of those operating within it. Thus, the Laureate network could be interpreted as being better structured for originality than is the non-Laureate network.

The network ties may give Laureates access to non-redundant information to a higher extent than the non-Laureates, who appear to gather more people with fewer connections into a modular community. This tendency among Laureates to connect-to-the-connected in turn facilitates originality—the coin of the realm in discovery-based science. It follows that highly creative researchers may seek one another out, perhaps not so much for cooperation (although that clearly occurs), but to stay abreast of what others are doing to ensure an advantageous position for originality relative to other high achievers. Investing social capital in network relationships (while 'costing' more in terms of time and attention than operating in a tight community) provides the pay-off of knowing what others are researching. This interpretation is consistent with findings about the Laureate network.

Finally, institutional theory suggests that advantages associated with prestige, attractiveness, and collaboration may accrue to the Laureates and to the Prize-winning network in a positive feedback loop [24]. Being awarded the Nobel Prize is a strong indicator of prior social capital, and it confers greater institutional legitimacy and social capital on the coauthor network as a whole. Given the higher rate of collaboration between Nobel Prize winners, membership in the Nobel network may confer greater institutional legitimacy. In other words, to some degree, the network itself is more visible, esteemed, and attractive simply because it contains more previously recognized and validated scientists. Having worked with a Laureate, in addition to conferring high-quality exchange of scientific knowledge, also confers a higher level of prestige on the collaborator. The coauthor of the Nobel Laureate then obtains a greater level of legitimacy in the scientific community, which impacts both the social capital of the individual and of the network overall.
