# Correction: Do Nobel Laureates Create Prize-Winning Networks? An Analysis of Collaborative Research in Physiology or Medicine

**DOI:** 10.1371/journal.pone.0136478

**Published:** 2015-08-19

**Authors:** Caroline S. Wagner, Edwin Horlings, Travis A. Whetsell, Pauline Mattsson, Katarina Nordqvist

There are errors in the Competing Interests statement on the published article. The correct Competing Interests statement should read: During the time of this research, KN served as the director of research at the Nobel Museum, Stockholm, Sweden.
